# Molecular beam epitaxy and properties of GaAsBi/GaAs quantum wells grown by molecular beam epitaxy: effect of thermal annealing

**DOI:** 10.1186/1556-276X-9-123

**Published:** 2014-03-17

**Authors:** Hajer Makhloufi, Poonyasiri Boonpeng, Simone Mazzucato, Julien Nicolai, Alexandre Arnoult, Teresa Hungria, Guy Lacoste, Christophe Gatel, Anne Ponchet, Hélène Carrère, Xavier Marie, Chantal Fontaine

**Affiliations:** 1 CNRS, LAAS, 7, avenue du Colonel Roche, Toulouse 31400, France; 2 Université Paul Sabatier, 118 route de Narbonne, Toulouse 31400, France; 3 Université de Toulouse, LPCNO, INSA-UPS-CNRS, 135 avenue de Rangueil, Toulouse 31400, France; 4 CNRS-CEMES, 29, rue Jeanne Marvig, Toulouse 31400, France; 5 Service Analyseur ionique, INSA, 135 avenue de Rangueil, Toulouse 31400, France; 6 Université de Toulouse, Toulouse 31400, France

**Keywords:** Dilute bismides, Molecular beam epitaxy, Heteroepitaxy, X-ray diffraction, Transmission electron microscopy, Photoluminescence

## Abstract

We have grown GaAsBi quantum wells by molecular beam epitaxy. We have studied the properties of a 7% Bi GaAsBi quantum well and their variation with thermal annealing. High-resolution X-ray diffraction, secondary ion mass spectrometry, and transmission electron microscopy have been employed to get some insight into its structural properties. Stationary and time-resolved photoluminescence shows that the quantum well emission, peaking at 1.23 μm at room temperature, can be improved by a rapid annealing at 650°C, while the use of a higher annealing temperature leads to emission degradation and blue-shifting due to the activation of non-radiative centers and bismuth diffusion from the quantum well.

## Background

Dilute bismuth alloys grown on GaAs attract more and more attention because of their peculiar electronic properties. Adding bismuth to GaAs efficiently decreases the gap energy of this semiconductor [1] through a change in its valence band properties and increases the spin-orbit interaction [2]. GaAsBi/GaAs quantum wells (QWs) are of interest with a view to fabricate laser diodes which could benefit from these properties, in particular from the higher spin-orbit splitting expected to lower the non-radiative carrier recombination due to Auger mechanisms [3]. Moreover, their emitting wavelength range could meet the requirements for infrared GaAs-based laser diodes as an alternative to low-temperature GaInAs/GaAs [4] and GaInAsN/GaAs [5] QWs. Besides, the properties of these III-V alloys are also very promising for photovoltaics [6]. Up to now, literature on GaAsBi has mainly been devoted to thick layers (see [7] and ref. herein), and only a few papers on the growth of quantum well structures have been published [8]. Here, we present the structural and optical properties of a GaAsBi/GaAs QW grown by molecular beam epitaxy (MBE) and discuss their change after rapid thermal annealing (RTA).

## Methods

GaAsBi quantum well structures were grown using a 32P RIBER (Bezons, France) MBE system. Substrates were pieces of a semi-insulating GaAs substrate soldered with indium on a silicon wafer mounted on the substrate holder to be loaded in the MBE system. The substrate thermocouple temperature for the molybdenum substrate holder was first calibrated by using a band edge thermometry system (BandIT). The control of the growing material was performed by reflection high-energy electron diffraction (RHEED). Our MBE system is designed to grow in the RIBER ‘optimal cell/sample oven’ geometry which leads to high thickness uniformity on 2-in. samples even though the 32P Riber MBE system is not normally designed to get high uniformity on these large surface areas. In such a geometry, substrate rotation is required to be used continuously during the growth, since the fluxes are not converging towards the substrate holder center.

QWs with different Bi contents and widths were grown, and the results presented here come from the QW emitting at the longer wavelength. They were grown after careful calibration of the growth conditions, the GaAs growth rate, i.e., the V/III ratio, the substrate temperature, and the Bi content, on thick GaAsBi layers. Note that we do not have any flux gauge in our MBE system, so the Bi control was carried out via the cell temperature.

The investigated QW sample consists of a 500-nm-thick buffer GaAs layer, the GaAsBi/GaAs QW, and finally a 100-nm-thick cap layer. After the growth of the buffer layer at 580°C, the temperature is lowered to 365°C, the value selected for the QW growth. The As cell valve opening is reduced in order to yield the As_4_ flux corresponding to a V/III atomic ratio close to unity, as needed for GaAsBi growth [9]; the As cell is a RIBER VCAS700 cracker (Bezons, France) one whose nose temperature is set to 650°C, thus mostly ejecting As_4_ species. At the same time, the Ga cell temperature is decreased to a value which leads to a low growth rate for GaAs, of the order of 0.25 ml/s. After substrate temperature cooling, care is taken to get temperature stabilization since this parameter plays a major role in Bi incorporation [7]. At this step, the Ga and Bi cell shutters are opened simultaneously. For the first period of growth, bismuth plays the role of a surfactant for the low-temperature-grown GaAs [9], until a (2 × 1) reconstruction of a bismuth-rich GaAs surface [10] is observed, the required condition for efficient incorporation of this element into GaAs [11]. Then, the bismuth element contributes to the formation of a GaAsBi QW. At the end of the QW growth, the Bi cell shutter is closed first. The Ga shutter is only closed once a 5-nm layer of GaAs has been grown; we have observed that the GaAs RHEED pattern deteriorates for a GaAs layer thickness higher than 5 to 10 nm, after the floating bismuth was incorporated in GaAs or desorbed [7]. The growth is then interrupted to heat the structure temperature to 520°C. Finally, a 30-nm-thick GaAs layer is grown at 0.7 ml/s at this temperature, and the 100-nm-thick barrier growth is completed while the temperature is raised to 580°C.

Once grown, the GaAsBi/GaAs QW structures were analyzed by stationary photoluminescence using the 514-nm line of an argon laser and a GaInAs photodetector. The quantum well emitting at 300 K at the longer wavelength, 1.23 μm, was selected. The sample was cleaved into pieces, which were subjected to *ex situ* RTA in an AnnealSys AS-One system (West Newbury, MA, USA). RTA was carried out in a nitrogen atmosphere during 30 s at annealing temperatures of 650°C, 700°C, 750°C, and 800°C. Samples were covered with a GaAs substrate during annealing in order to prevent surface degradation by arsenic desorption.

High-resolution X-ray diffraction (HR-XRD) was performed on the as-grown sample using a D8 Discover Bruker (Karlsruhe, Germany) equipment in order to determine its thickness and strain, from which we deduce its bismuth content. It was also used on the annealed samples to get insight into the evolution of their structural properties upon annealing. Secondary ion mass spectrometry (SIMS) with a CAMECA IMS-6f (Gennevilliers, France) was employed to measure the profile of the bismuth element within the structure for the as-grown and annealed samples. Primary Cs^+^ ions were accelerated at 3 kV, while the positive secondary ions were collected at 2 kV. Transmission electron microscopy (TEM) in conventional and high-resolution (HR) modes was carried out on the as-grown QW sample. A <110 > -oriented cross-sectional sample was thinned by mechanical polishing and ion milling. HR-TEM observations were performed at 200 kV on a TECNAI F-20 (E.A. Fischione Instruments, Inc., Export, PA, USA), equipped with a spherical aberration corrector tuned to avoid the delocalization effect at the interface and to achieve a 0.12-nm resolution.

Time-resolved photoluminescence (PL) spectroscopy was performed at room temperature on the as-grown and annealed samples. Optical excitation was provided by focusing 1.5-ps pulses generated by a mode-locked Ti-sapphire laser with 80-MHz repetition frequency. The laser wavelength was set to *λ*_exc_ = 795 nm with 20-mW incident power, focused to a 50-μm diameter spot at the sample surface. The signal was recorded using a S1 photocathode Hamamatsu streak camera (Hamamatsu, Shizuoka, Japan) with an overall time resolution of 8 ps. The signal was recorded in the high-energy side (1,120 to 1,220 nm) of the PL spectrum.

## Results and discussion

### As-grown quantum well structure

**Figure 1:**
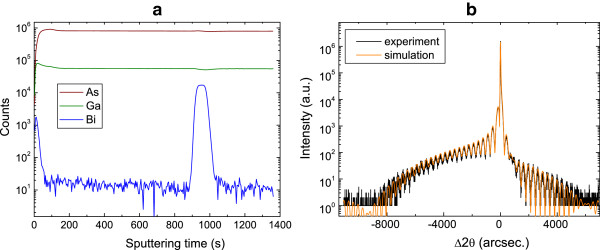
**SIMS profiles and X-ray diffraction scans.** SIMS profiles of the different elements (Ga, As, Bi) within the QW structure **(a)**; (004) X-ray diffraction *ω*/2*θ* transverse scans measured (black) and simulated (orange) for the QW structure **(b)**.

SIMS analysis was carried out on the as-grown sample in order to image the bismuth profile through the structure. Indeed, it is worth to recall that we opened bismuth with Ga and As for a longer duration compared to the one which should be applied for growth of a 7-nm QW. We postulated that the incorporation of the bismuth occurs when the right content of the element saturates the surface, giving rise to the (2 × 1) surface which promotes its incorporation as explained in [10]. The use of this method allows us to optimize the lower QW interface. We actually observe in Figure 1a for this QW that the bismuth content actually exhibits a step shape within the structure. We only observe a small Bi shoulder close to the lower interface which we associate to a slight incorporation of Bi before the quantum well. Moreover, no bismuth is observed to be incorporated in the subsequent 5-nm GaAs grown at low temperature while a As/Ga stoechiometric ratio was still being used. This supports our hypothesis that this element plays the role of a surfactant during the first stage of low-temperature GaAs:Bi growth and, once the bismuth cell was closed, during the low-temperature GaAs growth. Note that, contrary to the case of thick layers, the bismuth content cannot be directly inferred from the SIMS profile as the QW is too thin for SIMS to provide a direct correspondence with the absolute Bi content.

In order to determine the Bi content and QW thickness, HR-XRD analysis is used. As it can be observed in Figure 1b, the presence of thickness fringes and a narrow diffraction peak in the resulting 2*θ* transverse scan indicates that the grown material is of high quality. It allows us to accurately evaluate the thickness, content, and strain of the QW. The X-ray diffractogram was simulated. This calculation was done using the software provided by Bruker, with GaAs and GaBi lattice parameters taken equal to 0.56353 and 0.633 nm, respectively [12], and with the elastic constants of GaAs (*C*_11_ = 118.81 GPa and *C*_12_ = 53.8 GPa) for a structure under complete elastic tetragonal strain. For a thickness of 7.5 nm and a Bi content of 7%, we observe perfect agreement of the experimental rocking curve with the simulated one, further highlighting that the as-grown QW structure exhibits good quality structural properties.

**Figure 2:**
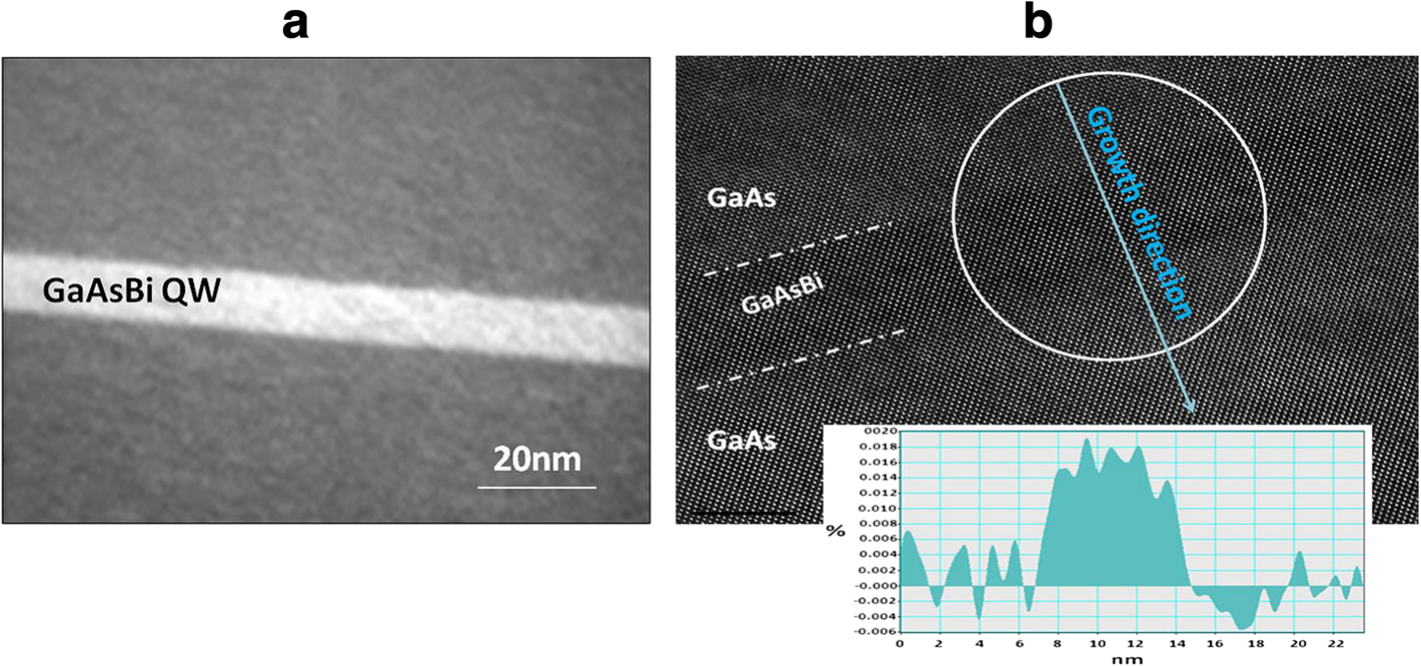
**TEM observation of a cross section of the QW structure.** Conventional **(a)** and high-resolution modes (inset: strain profile measured along the growth direction) **(b)**.

This is also supported by the TEM analysis. The abruptness and flatness of the lower and upper interfaces are confirmed. First, conventional TEM experiments reveal the absence of extended defects and insignificant roughness (Figure 2a). HR-TEM performed on various areas confirms on a more local scale the absence of dislocations (Figure 2b), indicating a full accommodation of the lattice misfit by elastic deformation. The strain related to a reference zone chosen in the GaAs buffer was determined with a spatial resolution better than 1 nm, by means of geometrical phase analysis of the HR-TEM images [13,14]. As shown by the profile along the growth direction (Figure 2b, inset), the out-of-plane strain *ϵ* can be considered as homogeneous in the quantum well. Following the linear elasticity and taking into account the fact that *ϵ* is measured related to a GaAs reference zone, *ϵ* is related to the misfit *f* through *ϵ* = (1 + 2*C*_12_/*C*_11_)*f*.

The average strain measured from HR-TEM is 0.016, but this value has to be corrected for surface relaxation effects: the strain before thinning is numerically estimated to be 10% to 20% larger [14].

Thus, after this correction, the estimated misfit is in the range of 0.0095 to 0.0105. Applying the Vegard’s law, this corresponds to a Bi amount of 7.8% to 8.8%, close to the content provided by HR-XRD. Different analyzed zones have given comparable values of strain. In addition, the measured QW thickness in Figure 2b is measured to be 7 nm, again close to the value estimated through HR-XRD.

Taking into account the values measured by HR-XRD and TEM, the assumption of late incorporation of the bismuth is valid. The thickness of the QW is thinner than the one which would have been obtained had the Bi atoms been directly incorporated as soon as the Bi cell was opened.

### Annealed QW samples

**Figure 3:**
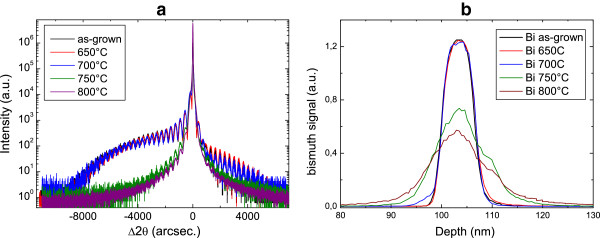
**X-ray diffraction scans and Bi SIMS profiles.** (004) X-ray diffraction *ω*/2*θ* transverse scans **(a)** and Bi SIMS profiles for the differently annealed samples **(b)**.

Figure 3a shows that the RTA of the QW structure leads to a drastic change in its X-ray 2*θ* transverse scan. The diffractogram is similar to the as-grown one except for the 650°C annealing temperature. At 700°C, a slight difference is observed, while for the two highest annealing temperatures, the background is observed to drastically evolve, which can be accounted for by a modification of the bismuth profile within the structure. Figure 3b shows how the SIMS Bi signal broadens with annealing. This analysis confirms that the Bi profile has become more distributed within the structure, as a result of its diffusion into the GaAs surrounding barriers for the two higher annealing temperatures. This Bi diffusion at high temperature is certainly supported by strain for this QW with a high Bi content. Indeed, Mohmad et al. [15] have shown that the optimal annealing temperature is affected by the local strain supported by the GaAsBi alloys due to the high atomic size of the Bi atom.

**Figure 4:**
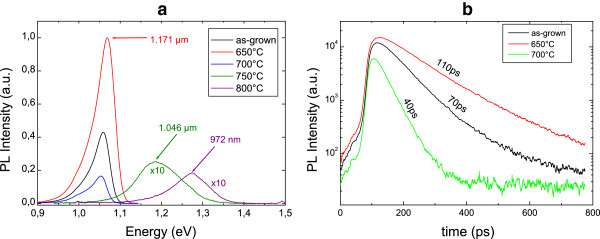
**Annealing effect on QW PL.** The 20-K PL spectra of the as-grown and annealed QW samples **(a)**; room temperature time-resolved PL carrier decay time for the as-grown QW and those annealed at 650°C and 700°C **(b)**.

As a consequence, the QW optical properties are greatly affected by the RTA treatment. Figure 4a shows the evolution of the 20-K PL spectra for different annealing temperature conditions. The QW emission wavelength is unchanged for the 650°C and 700°C RTA, but it strongly blue-shifts at higher RTA temperatures, due to the Bi diffusion out of the annealed QW. Moreover, only the 650°C RTA improves the emission intensity compared to the as-grown sample. For higher temperatures, a rapid reduction of the PL intensity is found. Additionally, at room temperature, the PL emission of the 650°C RTA QW is found to be better than the one of the as-grown QW, while no PL emission is visible for the annealed samples at the 750°C and 800°C. RTA is known to improve structural properties of materials. In the case of GaAsBi thin layers, a decrease of the density of localized defects, due to bismuth aggregates or alloy disorder, has been claimed to occur during annealing by Mohmad et al. [15] and Mazzucato et al. [16].

The increase of the carrier decay time in the time-resolved luminescence analysis measured for the 650°C annealed sample, in Figure 4b, also supports its better crystallinity, in agreement with the improved emission recorded under CW photoluminescence analysis. On the contrary, for annealing treatment at temperatures higher than 700°C, the room temperature carrier decay time like the other properties of this annealed QW degrades with respect to the as-grown QW, showing again that a too high annealing temperature leads to the activation of additional non-radiative centers.

## Conclusions

We have shown that a 7% Bi GaAsBi quantum well grown by molecular beam epitaxy, emitting at 1.23 μm at room temperature is completely elastically strained and exhibits good structural properties, with a uniform thickness, sharp interfaces, and the absence of extended defects. Annealing this QW leads to bismuth out-diffusion as soon as the applied annealing temperature is higher than 650°C. At the latter annealing temperature, the photoluminescence emission and decay time at room temperature are all improved.

## Competing interests

The authors declare that they have no competing interests.

## Authors’ contributions

HM and CF grew and annealed the samples. PB studied their PL properties. AA took in charge of the X-ray diffraction analyses. SM, HC, and XM carried out the TRPL experiments. GL settled the MBE system for bismide growth. TH performed the SIMS analyses. JN, CG, and AP carried out the TEM analyses. All authors interact on the results and on their analysis, and read and approved the final manuscript.

## Authors’ information

Hajer Makhloufi is a PhD student at LAAS. Poonyasiri Boonpeng is a TECHNO Tempus Erasmus Post-doctorate at LAAS. Simone Mazzucato is a visitor researcher at LPCNO. Hélène Carrère holds an assistant professor position at INSA and is a researcher at LPCNO. Julien Nicolai is a CNRS post-doctorant at CEMES. Alexandre Arnoult and Guy Lacoste are CNRS engineers at LAAS. Teresa Hungria is an engineer at INSA. Christophe Gatel (assistant professor at Université Paul Sabatier) and Anne Ponchet (Research Director at CNRS) are researchers at CEMES. Xavier Marie is a professor at INSA and is a researcher at LPCNO. Chantal Fontaine (Research Director at CNRS) is a CNRS researcher at LAAS.
